# Safety and Efficacy of Pneumococcal Vaccination in Pediatric Nephrotic Syndrome

**DOI:** 10.3389/fped.2019.00339

**Published:** 2019-08-13

**Authors:** Shamitha Thishakya Goonewardene, Calyn Tang, Loh Teng-Hern Tan, Kok-Gan Chan, Prithvy Lingham, Learn-Han Lee, Bey-Hing Goh, Priyia Pusparajah

**Affiliations:** ^1^Medical Health and Translational Research Group, Jeffrey Cheah School of Medicine and Health Sciences, Monash University Malaysia, Subang Jaya, Malaysia; ^2^Novel Bacteria and Drug Discovery Research Group, Microbiome and Bioresource Research Strength Jeffrey Cheah School of Medicine and Health Sciences, Monash University Malaysia, Subang Jaya, Malaysia; ^3^Biofunctional Molecule Exploratory Research Group, School of Pharmacy, Monash University Malaysia, Subang Jaya, Malaysia; ^4^Institute of Biomedical and Pharmaceutical Sciences, Guangdong University of Technology, Guangzhou, China; ^5^Division of Genetics and Molecular Biology, Institute of Biological Sciences, Faculty of Science, University of Malaya, Kuala Lumpur, Malaysia; ^6^International Genome Centre, Jiangsu University, Zhenjiang, China; ^7^Health and Well-being Cluster, Global Asia in the 21st Century Platform, Monash University Malaysia, Bandar Sunway, Malaysia

**Keywords:** nephrotic, pediatric, pneumococcal, vaccination, efficacy

## Abstract

Nephrotic syndrome affects both children and adults. Idiopathic nephrotic syndrome is reported to be one of the most frequent renal pathologies in childhood. Nephrotic children are at high risk for severe pneumococcal infections as one of the life-threatening complications of nephrotic syndrome due to involvement of the immunosuppressive regimen and the acquired immune deficiency induced by nephrotic syndrome including decreased plasma IgG and low complement system components. Aiming to prevent pneumococcal infection is of paramount importance especially in this era of ever-increasing pneumococcal resistance to penicillins and cephalosporins. The pneumococcal vaccines currently available are inactivated vaccines—the two main forms in use are polysaccharide vaccines and conjugated vaccines. However, the data supporting the use of these vaccines and to guide the timing and dosage recommendations is still limited for nephrotic children. Thus, this review discusses the evidences of immunogenicity and safety profile of both vaccinations on nephrotic patients as well as the effect of nephrotic syndrome treatment on vaccine seroresponses.

## Introduction

Nephrotic Syndrome (NS)—an important kidney disorder affecting both children and adults (1)—is characterized by heavy proteinuria leading to hypoalbuminemia and oedema (2). NS has a worldwide prevalence of 16 cases per 100,000 children, while incidence has been recorded to be 2–7 per 100,000 children (3). NS is most commonly seen among school aged children and adolescents, but there are two subsets based on age of presentation—Congenital Nephrotic Syndrome (presentation within first 3 months of life) and Infantile Nephrotic syndrome (presenting between 3 months to 1 year of age) (4). Among children with nephrotic syndrome, only a minority (20–33%) have a single episode, while the majority have multiple relapses. Male gender and young age at initial presentation have been identified as risk factors for frequent relapses (5).

NS can broadly be divided into two groups based on underlying etiology: primary (idiopathic) and secondary, with primary being the dominant type. Primary or idiopathic nephrotic syndrome can be further subclassified based on histological characteristics or based on steroid responsiveness. Minimal change disease represents the main histological subtypes identified for idiopathic Nephrotic Syndrome (INS) in childhood with the less common subtypes being focal segmental glomerulosclerosis and membranous nephropathy. INS is the most common form of disease in childhood and clinically, it is subdivided further based on response to steroid therapy into steroid-sensitive (SSNS) and steroid resistant (SRNS) forms. The SSNS has further subgroups including frequent relapsers and steroid dependent nephrotic syndrome (SDNS). The majority of patients (80–90%) respond to steroid therapy within 4 weeks of administration (SSNS) while SRNS is defined by persistent proteinuria in spite of 4 weeks of prednisolone therapy, while for SDNS, the hallmark is relapses while on steroid therapy or within 2 weeks of discontinuation (2).

From a clinical point of view, the classification of INS according to its response to steroid therapy has many implications, particularly with reference to increased risk of severe infection as steroids are immunosuppressive thus increasing the risk of infection. SRNS is associated with heavy proteinuria which is also linked to higher risk of infection, possibly through urinary losses of immunoglobulins (6, 7).

While there is a lack of definitive evidence regarding the mechanisms of pathogenesis of INS, there are a number of theories linking it to immune system dysregulation. This has implications both in terms of susceptibility to infection as well as possibly suboptimal response to vaccines. One theory implicates T cell dysfunction leading to cytokine release which results in increased glomerular permeability (3). It has also been suggested that immune system dysfunction may result in increased levels of circulating factors that may cause altered podocyte structure/function, resulting in proteinuria (8). The possibility of B cell involvement has been suggested based on patients achieving remission following administration of rituximab, an anti CD20 antibody (9).

*Streptococcus pneumoniae* is a major burden of infectious disease globally. In 2005, WHO estimated that 1.6 million people die of pneumococcal disease every year. This includes 0.7–1 million children below the age of 5 most of whom live in developing countries; while in developed countries the major disease burden is carried by children aged <2 years old and the elderly. Any immunocompromising condition increases the likelihood of contracting pneumococcal disease (10). The growing resistance of *S. pneumoniae* to commonly used antibiotics underlines the urgent need for vaccines to be used to control pneumococcal disease.

The immune system dysregulation that is part of the pathophysiology of NS as well as the immunosuppressive drugs that are the standard therapy for NS results in children with NS being at increased risk of infections—the most serious among these being peritonitis and sepsis as they carry a high morbidity and mortality. One of the most common organisms associated with these in NS patients is *S. pneumoniae*. Although the pneumococcal vaccination has demonstrated significant reduction in disease burden, there is often significant confusion among medical professionals regarding the protocol for vaccinating children with NS against pneumococci, resulting in many of these children not being appropriately vaccinated. Our review will focus on the efficacy and safety of pneumococcal vaccination in children with NS. We also discuss the mechanisms of immunosuppression in NS as well as the current recommendations for pneumococcal vaccinations.

## Pneumococcal Infection in Children With Nephrotic Syndrome

Children with NS are prone to developing significant bacterial infections; and although the incidence of infections in NS has decreased in advanced countries, they are still a major problem in developing countries. Data shows that sepsis remains one of the main causes of death in children with NS while peritonitis is one of the major causes of morbidity and mortality in NS patients, and the main organism responsible for primary peritonitis in NS patients is *Streptococcus pneumoniae* (11).

From April 1993 to December 1997, Chang Gung Children's Hospital in Taiwan had 452 admissions of 231 children with nephrotic syndrome. There were 10 episodes of sepsis and 8 episodes of peritonitis, with 14 microorganisms cultured. Out of the 10 episodes of sepsis, 4 were due to *S. pneumoniae* and 2 of these 4 children died; there were no deaths recorded for the other organisms. There were no cases of peritonitis due so *S. pneumoniae* in this data (12). This data was all collected prior to the introduction of pneumococcal vaccinations in Taiwan where PPV was first licensed in 1998 but rarely used before 2001, while PCV7 was first introduced in late 2005 (13).

Krensky et al. (14) noted that between 1970 to 1980, in a retrospective review of 351 pediatric INS cases, there were 24 episodes of peritonitis among 19 patients, with >50% of the cases being due to *S. pneumoniae*. This was based on data from Boston and PPV was first licensed in the USA in 1977.

A 5-year multicentre study in Turkey involving 268 children with SSNS noted only 8 episodes of peritonitis among 7 patients (incidence of 2.6%) (15). Among the cases of peritonitis noted, the majority were caused by *Streptococcus pneumoniae*, with others being attributed to Gram negative bacteria (15). The 7-valent conjugate pneumococcal vaccine (PCV7) was introduced by the Turkey National Immunization Program in 2008 and replaced by the PCV13 in 2011 (16). The majority of these studies were done prior to the large scale introduction of PPV, and well before PCV in 2000.

We were unable to locate data specifically mentioning frequency of *S. pneumoniae* infection in NS patients after the introduction of pneumococcal vaccinations, however global data do suggest that the vaccinations significantly reduce the disease burden which highlights the critical nature of preventing pneumococcal infection in NS patients by appropriately vaccinating these patients.

## Pathophysiology of Increased Susceptibility to Infection in NS and Implications of Immune Response to Vaccination

**Figure 1:**
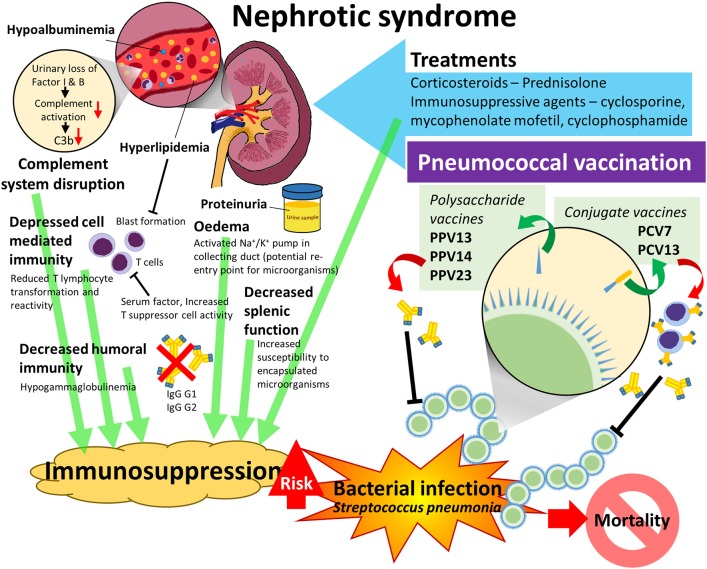
Predisposing factors to pneumococcal infection in Nephrotic Syndrome and pneumococcal vaccination as a strategy to reduce the risk of pneumococcal infection.

Immune deficiency is part of the clinical picture of NS and is believed to be multifactorial, but there is limited data regarding the precise nature and contribution of these factors. Figure 1 illustrates the multiple pathophysiological elements of NS that contribute to an increased risk of pneumococcal infection in these patients. An understanding of the theories related to immunosuppression in NS are key to this review as this is both the reason for increased susceptibility to invasive pneumococcal infection hence the strong need for vaccination; but it also may impact on the vaccine's efficacy in NS patients given that this requires a healthy immune system that is able to mount a response upon exposure to bacterial antigen. This conundrum reflects part of the confusion surrounding how and when to give pneumococcal vaccinations to pediatric NS patients as although the protection from the vaccine is very much needed, there is confusion regarding whether it should be given and if so, at which point in the disease process it should be given.

**Figure 2:**
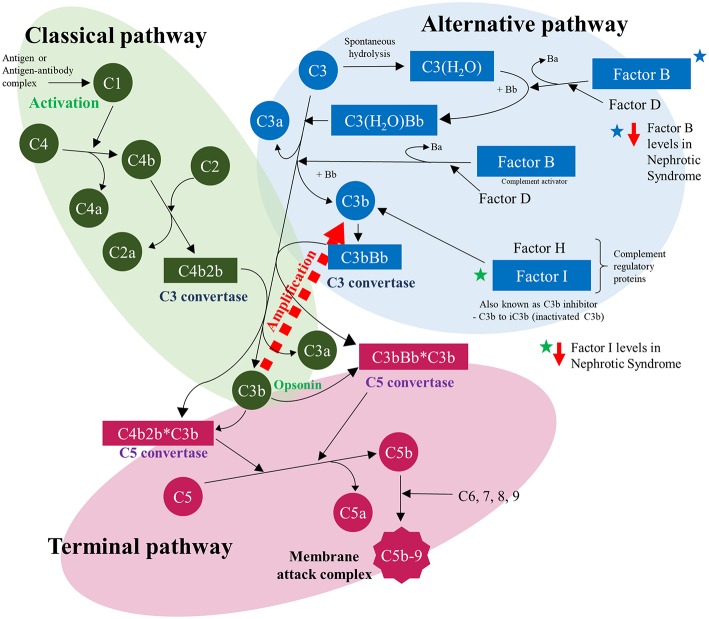
Complement cascade for classical and alternative pathways illustrating the deficiencies in the complement system in Nephrotic Syndrome.

Disruption of the complement system is one of the better understood causes of immunosuppression in nephrotic syndrome. The complement system is a key component of innate and adaptive immune responses and consists of two arms: the classical and the alternative. Several studies have demonstrated deficiencies in Factor I and Factor B—two factors that control C3 which is a key mediator of both the classical and alternative complement pathways. When activated by either the classical or alternative pathway, C3 is broken down into C3a and C3b, with C3b being an active form which is a potent opsonin. Various factors control the levels and activity of Factor C3b—including Factor I and Factor B. Factor I, also known as KAF, functions as a C3b inhibitor; whereas factor B combines with C3b. Deficiencies in Factor I and Factor B have been demonstrated to be associated with severe infections (17, 18). In nephrotic patients, reduced levels of Factor I and Factor B have been reported (19, 20). Figure 2 shows an overview of the complement cascade and where the alterations occur in NS patients which then predisposes to infection and also possibly leading to concerns regarding efficacy of vaccines.

As early as 1977, it was reported that increased incidence of severe infection in patients with NS may be attributed to urinary losses of Factor B. The disturbance to the alternative complement pathway increases the susceptibility of nephrotic patients to pyogenic infection, particularly encapsulated microorganisms like *S. pneumoniae* due to the impaired complement dependent opsonisation (3, 21, 22).

The complement system has also been shown to have a key role in the ability to mount an effective response to vaccines, though it appears to have a larger role in response to polysaccharide vaccine compared to conjugated vaccines (23).

Depression of cell mediated immunity is another postulated mechanism of immunosuppression in nephrotic syndrome. It has long been accepted that there is a change in the T-lymphocyte response in NS which causes increased production of permeability factors, changing the function of key podocyte proteins thus causing the proteinuria which is the hallmark of nephrotic syndrome (3). Fodor et al. suggested that a circulating serum factor is responsible for a reduction in T-lymphocyte function in childhood minimal change nephrotic syndrome (24). The study reported that even though T cell numbers remained in the normal range, there was a marked reduction in lymphocyte transformation during active disease. Nephrotic plasma was also found to inhibit the reactivity of normal lymphocytes, possibly due to the hyperlipidaemic conditions (characteristic of NS) which inhibit blastic transformation. The authors also proposed that decreased cell mediated immunity may be the effect of increased suppressor T cell activity (24). Taube et al. also demonstrated diminished lymphocytic transformation in NS due to minimal change disease, focal segmental glomerulosclerosis, and membranous nephropathy (25). However, the findings of Taube et al. suggested that the reduced lymphocyte response is likely secondary to the nephrotic state rather than due to a circulating factor (25). As shown in Figure 1, T cells are a key part of the immune response to conjugated vaccines, and deficits in their function may impair the immunogenicity of the vaccine.

Besides cell mediated immunity, alteration in humoral immunity is also believed to contribute to the immune deficiency associated with NS. It has been shown that during relapse periods of SSNS, there is hypogammaglobulinaemia involving the reduction of three IgG subclasses- Ig G1, Ig G2, and IgG3- placing patients at higher risk of bacterial and viral infection. However, interestingly, only Ig G2 remained significantly low throughout the remission period, suggesting it is the main factor of persistent hypogammaglobulinaemia during remission (26). This also raises the question of whether even if the body can mount an immune response whether the protective antibodies are then lost in the urine, thought to date data on this is lacking.

The increased susceptibility to infection in NS patients raises the question of whether they would benefit from immunizations, however the altered immune function in NS patients also raises significant questions about the efficacy of vaccinations in this population in terms of the immune system ability to mount an adequate antibody response.

Oedema is yet another potential factor contributing to risk of infection in nephrotic syndrome (27). Oedema is a hallmark of NS and occurs due to the hypoalbuminemia and increased capillary permeability—this results in intravascular volume depletion which triggers compensatory mechanisms to reabsorb sodium and water, one of which is activation of the sodium-potassium pump in the collecting duct of the kidney which increases the possibility of entry of infections agents and transmission of infection (27, 28). Infective complications have been shown to be reduced when the duration of oedema is lessened, however whether oedema really predisposes to infection or is just simply a marker of serious and unresponsive disease is uncertain (27, 29).

Defective splenic function is another possible factor for the increased susceptibility to encapsulated organisms in patients with nephrotic syndrome. Mcvicar et al. demonstrated that among nine children with primary NS, 4 had decreased splenic function and that particular patient population had 6 episodes of bacterial infection, suggesting a link between splenic hypofunction and increased risk of bacterial infection (30). However, Berns et al. reported that impaired splenic function is unlikely to be a major cause in the predisposition of NS patients toward bacterial infection (31).

Lastly, iatrogenic causes do contribute to NS related immunosuppression due to the cytotoxic agents used as treatment. A meta-analysis investigating the use of cyclophosphamide and chlorambucil as treatment of relapsing NS in children found that among the subjects treated with either drug, 1/3 of them were shown to have leukopenia while 1.5 and 6.8% treated with cyclophosphamide and chlorambucil, respectively, developed severe bacterial infections (32). There have been doubts regarding efficacy of vaccines given to nephrotic patients who are on high dose steroids as their immunosuppressive effects may also diminish the immune response to the vaccine. Data on this is presented in **Tables 2**, **3**.

There are significant numbers of both viral and bacterial infections documented in children with NS. However, bacterial infections tend to be more worrying as they represent the main cause of mortality among children with NS; and the predominant culprits for these bacterial infections are *S. pneumoniae, Escherichia coli* and *Haemophilus* sp. (45). The most common among these causative agents was *S. pneumoniae* at 38 and 50% as demonstrated by Krensky et al. and Gorensek et al., respectively (14, 29, 46). Given that infection with *S. pneumoniae* is largely vaccine preventable, vaccination against pneumococcus is recommended for pediatric NS patients (47). There are at present 2 options available—a 13 valent conjugate vaccine and a 23-valent polysaccharide vaccine, and this review aims to explore the evidence regarding the effectiveness and safety of pneumococcal vaccination in children with NS.

## Pneumococcal Vaccinations—Their Development and Mechanism of Action

The first ever attempt to vaccinate humans against pneumococcus took place in Africa in 1911 using a whole cell vaccine. Advances in understanding germ theory led to typing of pneumococci then the isolation of capsular polysaccharides in 1916. This then paved the way for the creation of the first quadrivalent pneumococcal polysaccharide vaccine (PPV) in 1944–1945 which did show promising results, then a hexavalent vaccine was developed in 1947. However, at this point, clinicians preferred to treat with antibiotics rather than vaccinate until it was recognized that antibiotic resistance was becoming a concern with continued high case fatality rates in spite of treatment; thus, efforts were reintensified to create effective vaccines. This then led to a to 14 valent PPV being licensed in 1977 and this was followed by the 23 valent pneumococcal vaccine which was first introduced in the United States in 1983 (48). Polysaccharide vaccines act via a T-cell independent mode of B-cell stimulation which is not able to generate a memory effect; and pneumococcal polysaccharide vaccines are poorly immunogenic in children younger than 2 years of age. This is of concern as 80% of invasive pneumococcal disease occurs in children under the age of 2 (49).

**Figure 3:**
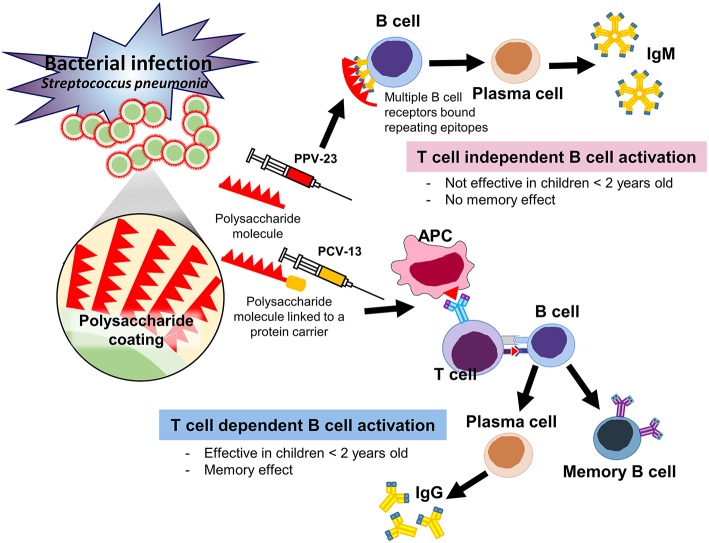
Mechanisms of immunogenicity of PPV vs. PCV.

To improve the immune response to the capsular polysaccharide in young children, third-generation vaccines in which capsular polysaccharides are conjugated to one of several different proteins were developed. The first such vaccine to be registered by the FDA for use in infants and children in February 2000 was a 7 valent pneumococcal conjugate vaccine (PCV) named Prevnar 7 (50). Following this a 13 valent pneumococcal conjugate vaccine was licensed in March 2010 (51), and now a 13-valent PCV is available. Conjugate vaccines act via stimulation of T cells and consequently tend to have a superior antibody response, maintenance of antibody levels for a longer duration and induction of immunological memory (52). In addition, conjugate pneumococcal vaccines have been shown to have excellent immunogenicity in children below 2 years of age (49, 53, 54). Figure 3 depicts the differences between PPV and PCV in terms of their structure and the immune response.

Pneumococci are divided about 90 distinct pneumococcal serotypes based on differences in the polysaccharide composition of the capsule. The spectrum of prevailing capsular types varies with age, time and geographical region, although common serotypes are consistently identified throughout the world. Globally, about 20 serotypes are associated with >80% of invasive pneumococcal disease occurring in all age groups, the 13 most common serotypes cause at least 70–75% of invasive disease (10).The vaccines have always targeted the most prevalent strains causing invasive disease and since their introduction, there have been tremendous reductions in invasive pneumococcal disease due to strains included in the vaccines (55). A Cochrane review of conjugate pneumococcal vaccines reported that the pooled vaccine efficacy was 80% (95% CI 58–90%) against vaccine-type disease and 58% (95% CI 29–75%) against all serotype invasive disease in children under 2 year (56). However, the rates of infection due to strains not included in the vaccines has risen (55). Aiming to prevent pneumococcal infection is of paramount importance especially in this era of ever increasing pneumococcal resistance to penicillins and cephalosporins.

## Current Recommendations for Pneumococcal Vaccination

### In Healthy Children

In a 2007 position paper, the WHO strongly recommended that pneumococcal vaccination be included in national childhood immunization programme (10). As of 2018, 142 countries had introduced the pneumococcal vaccination as part of their national immunization program (57).

For PCV administration to infants, WHO recommends 3 primary doses (the 3p+0 schedule); or 2 primary doses plus a booster (the 2p+1 schedule). If the 3p+0 schedule is used, vaccination can be initiated as early as 6 weeks of age with an interval between doses of 4–8 weeks, with doses given at 6, 10, and 14 weeks or at 2, 4, and 6 months, depending on programmatic convenience. If the 2p+1 schedule is selected, the 2 primary doses should be given during infancy as early as 6 weeks of age at an interval preferably of 8 weeks or more for the youngest infants and 4–8 weeks or more between primary doses for infants aged ≥7 months. One booster dose should be given between 9 and 15 months of age. In choosing between the 3p+0 and 2p+1 schedules, countries should consider locally relevant factors including the epidemiology of pneumococcal disease, the likely coverage, and the timeliness of the vaccine. This is summarized in Table 1.

**Table 1:** Summary of WHO recommended pneumococcal vaccination schedules with PCV 13 for all children <2 years of age.

**Pneumococcal vaccine regimen**	**Dose 1**	**Dose 2**	**Dose 3**	**Booster**
2 + 1	Within 6 months, at least 8 weeks apart; starting as early as 6 weeks		NA	Between 9–18 months
3 + 0	Within 9 months, at least 4 weeks between doses; starting as early as 6 weeks; to be completed by age 2 years			NA
3 + 1	2 months	4 months	6 months	12–15 months

*In developed countries, 3 + 1 is recommended but countries are advised to choose the regimen best suited to their region and accessibility to healthcare*.

If this schedule is adhered to all children will be protected from the main pneumococcal strains by the age of 6 months, and given that the majority of cases of NS occur between the ages of 2 and 6 years with only a very small fraction having congenital NS and infantile NS, the vast majority of children with NS will already have protection against pneumococcal infection at the time of first presentation.

### Recommendations for Immunocompromised Children

It is recommended that immunocompromised children aged 2–18 who have completed the immunization with PCV 7 or PCV 13 should receive a dose of PPV 23 in view of the increased spectrum of coverage against more serotypes (58).

In the UK, specific recommendations exist for NS where children with INS are offered an additional dose of PPV when they are above 2 years of age (59).

**Figure 4:**
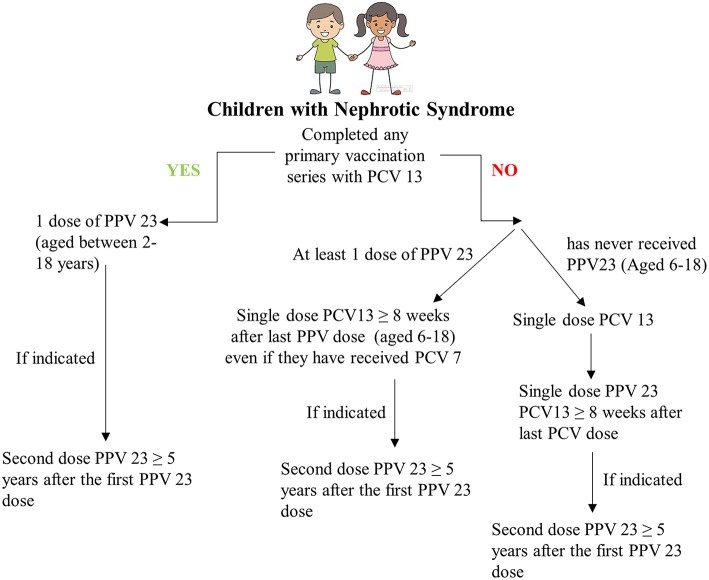
Recommended pneumococcal immunization pathway for children with Nephrotic Syndrome.

ACIP guidelines for immunocompromised children between the ages of 6 and 18 are that PPV naïve children receive a first dose of PCV 13 followed at least 8 weeks later by a dose of PPV 23 and then another dose of PPV23 at least 5 years later. Those vaccinated with PPV23 should receive a single dose of PCV 13 at least 8 weeks after the last PPV23 dose, even if they had previously received PCV7. Children under the age of 10 with ongoing INS may require revaccination 5 years after this initial regimen (60). This is summarized in Figure 4.

Of note, in USA, the cost per dose in the private sector for PPV13 is US$100.19 while the cost of PCV13 is US$188.26 (61). Although the PCV is significantly more expensive, the fact it confers protection on the vulnerable group of <2 year olds justifies the cost. It is worth noting that even though the vaccines have been introduced in a country, unless they are included in the national immunization program, the cost of having the vaccine administered privately results in low uptake among the population. In recognition of this fact, however, in Gavi 73 countries eligible for financial aid, PCV 13 vaccine can be purchased at US$2.90 per dose as of 2019, a price reduction of 5cents compared to 2018, making the vaccine affordable in an effort to reduce disease burden.

Smith et al. (62) compared the cost effectiveness of PCV 13 vs. PPV 23 and showed that PCV13 vaccination was favored compared with PPSV23, but the analysis was sensitive to assumptions about PCV13 effectiveness against non-bacteremic pneumococcal pneumonia and the magnitude of potential indirect effects from childhood PCV13 on pneumococcal serotype distribution. This work used pneumococcal disease cases prevented and incremental costs per quality-adjusted life-year (QALY) gained as the outcome measured. Delgleize et al. (63) showed that the price increase associated with converting PCV 7 to PCV 13 in the UK would be cost effective in terms of reducing cost associated with invasive pneumococcal disease saving 734 QALYs as well as £3.68 million to the National Health Service (NHS). Although the cost of PCV13 is prohibitive, a recent study by Chen et al. (64) published in the Lancet Global Health estimate that global PCV13 use could prevent 0.399 million child deaths (95% credible interval 0.208–0.711 million) and 54.6 million disease episodes (51.8–58.1 million) annually. Their calculations showed that the cost of vaccinations would be offset by societal cost savings of $2.64 billion (2.13–3.28 billion).

In high risk cohorts such as the immunocompromised pediatric NS patients, even those who have completed their primary regimen (as long as they are >2 years) are recommended to receive a dose of PPV23 which has the advantage of covering a greater number of strains even though it lacks the advantage of a memory or booster effect. However, the common clinical dilemma is the key question surrounding vaccine efficacy in NS children i.e., whether or not the vaccine is actually effective in active disease or if disease related or treatment related immunosuppression actually renders the pneumococcal vaccine ineffective.

## Efficacy of Pneumococcal Vaccine in NS Patients

Pneumococcal polysaccharide vaccine (PPV) has long been used for the prevention of invasive pneumococcal disease in children with nephrotic syndrome, and more recently PCV has been used since its introduction in the year 2000. Even though its effectiveness in evoking an antibody response has been well-demonstrated by several published studies (6, 33, 34, 37), there has been some controversy regarding its actual benefit as patients with NS have been reported to develop severe pneumococcal disease despite vaccination. There is also a lack of agreement as to the optimal timing of administration with reference to stages of NS (7, 34, 37, 65). The data from various studies looking at the efficacy of vaccines in nephrotic patients is summarized in Table 2 (for PPV) and Table 3 (for PCV).

**Table 2:** Summary of trials of PPV efficacy in pediatric Nephrotic Syndrome population.

**Study group, follow up duration per patient**	**Study year**	**Primary aim**	**Number of subjects (Intervention group and Control/comparison group)**	**Age group (mean/ median)**	**Type of vaccine used**	**Steroids/ medications intake, dosage, duration (if applicable)**	**Results**	**Reference**
Patients with NS, 6 weeks	1978	To assess the antibody responses against capsular antigens To investigate if suppressor T cells have a role in the mechanism of lipoid nephrosis.	NS group, *n* = 19; Age-matched and sex-matched control group, *n* = 23		PPV13	NIL	•The initial geometric mean titer (GMT) of antibody were lower in NS patients compared to control group. •Antibody concentration of NS group rises significantly (> or equal to 40% except against strain 19) and becomes comparable to the GMT in control group. •The stage of NS (Relapse or remission) as well as presence or absence of steroid therapy were found to have no effect on the antibody response.	(33)
Children with SSNS, 6 weeks	1982	To study serological response to pneumococcal vaccine in NS patients on steroid therapy	Daily prednisolone group, *n* =5; Alternate day prednisolone group (vaccine given on the day without steroids intake), *n* = 15		PPV14	If given, 1 to 2 mg/kg/day	•Pneumococcal vaccination provides good protection against strains covered by the vaccine even with concurrent steroid administration. •3 patients developed peritonitis - two cases were due to strains not covered by the vaccine (6b and 10a); the typing of the causative agent of peritonitis was not carried out for the third case	(34)
Children with INS, 5 weeks	1982	To assess the antipneumoccocal capsular antibody concentration and immunologic competence in SSNS and SRNS; analysis also performed in relation to steroid/immunosupressive therapy at time of vaccination	SSNS group, *n* = 27 (13 on corticosteroids at time of vaccination; 12 not on steroids; 2 on steroids + immunosuppressive therapy); Steroid resistant NS group, *n* = 6 (1 on steroids at time of vaccination; 5 not on steroids); Age-matched control group, *n* = 12	INS, 2–18 years (mean: 9.3), control group, 5 to 15 years (mean: 9.3)	PPV14	NIL	•All patients in SSNS (steroid receiving) group achieved geometric mean concentration (GMC) of > 200 ngN/ml (nanogram antibody nitrogen per milliliter) for all strains except strain 19F post vaccination. •The GMC of antibodies for all other strains, barring strain 12 F were similar to the increase shown by the control group. •Patients in SSNS (non-steroid receiving) group reached GMC of > 200 ngN/ml except for strain 6A and also show a lower increase of antibodies titres against strain 12F compared to control group. •Steroid resistant NS group (non-steroid receiving), despite achieving at least a 2 fold increase in antibody level post vaccination, the grand antibody concentration of 48 ngN/ml remained significantly lower than the controls (492 ngN/ml). Furthermore, only 1/ 12 patients in SRNS group‘ had GMC > 200 ngN/ml.	(6)
Children with NS, 5 years	1984	To investigate the persistence of antibody concentrations *in vivo* 5 years after initial vaccination.	Total patients =16 Minimal change nephrotic syndrome (MCNS) group, *n* = 9; Non-MCNS group (focal sclerosis, membranoproliferative glomerulonephritis or IgM nephropathy), *n* = 7; Non-vaccinated nephrotic patients control group, *n* = 20	MCNS group, 2.5–16 years, Non MCNS group, 5 to 16 years	PPV14	cyclophsophamide given to some patients: 2–3 mg/kg, 8 weeks	•MCNS group had GMT of antibodies greater than the protective value, 300 ngN/mL at the end of the study, whereas non-MCNS had GMT lower than the protective value. IgG level of those with non-MCNS is lower than the normal level as well and ranged from 180 to 600 mg/dL. •Among vaccinated patients, pneumococcal peritonitis only manifested in the patient with focal sclerosis who had the lowest antibody titer, 27 ngAb/mL; compared to 7/20 in the non-vaccinated control group who developed peritonitis (*p* < 0.05).	(35)
Children with SSNS, 12 months	1986	To investigate the anti-pneumococcal capsular polysaccharide antibody concentration 1 year after vaccination.	*N* = 25 -Non-relapsers (group 1) and relapsers (group 2) (at least on relapse but sera was taken during remission period)		PPV14		•The rate of decline in anti-pneumococcal capsular antibody concentration was more rapid in relapsers, (by 2.9%) compared to non-relapsers. •50% of all patients had <300 ngAbN/mL against types 4, 6A, 7F 8, and 19F Sera 1 year after vaccination	(36)
Children with active NS; 4 weeks	1988	To assess the IgM and IgG antibody response toward pneumococcal serotype 3 and 19 in patients with active NS.	Active nephrotic syndrome group, *n* = 11; Healthy adult controls group, *n* = 15	active nephrotic group, 2 years 5 months to 20 years 10 months, adult group; age not mentioned	PPV14, 50 micrograms or PPV23, 25 micrograms during active disease	NIL	•Prior to immunization, antibody levels against PS3 and PS19 were measured. For PS 3: IgM antibody concentration was found to be higher (*p* < 0.05) and IgG level was lower (*p* < 0.0025) among nephrotic patients compared to controls. For PS19: no significant difference in both IgM and IgG antibody concentration between patients and control group. •4 weeks post immunization, no significant difference in IgM level of both groups against both serotypes. However, IgG levels post immunization were significantly lower (*p* < 0.001) in nephrotic patients compared to controls. Control group showed remarkable elevations in IgG antibody titer against both serotypes, whereas nephrotic patients demonstrated significant increase in IgG antibody only against PS3 and not PS19. •A significant correlation between serum albumin level and type 3 (*p* < 0.01) and type 19 (*p* < 0.05) IgG antibody titer levels was noted. •IgG antibodies were detected in the urine of nephrotic patients but not the controls; IgM was non-detectable in the urine of both nephrotic patients and controls groups.	(7)
Children at increased risk of pneumococcal infection –children with: NS, splenectomy, recurrent bronchitis; 4 weeks	1995	To study the immunogenicity and safety of the vaccine in healthy as well as immunocompromised children.	Healthy subjects group, *n* = 21; NS group, *n* = 48 (data not included for other groups)	NS children, 1.8–18.4 (mean: 8.7 years), Healthy children, 3.0 to 13.8 years (mean: 8 years),	PPV23	NIL	•Pre-vaccination antibody levels for strains (6B, 9V, 14) as well as combined GM antibody concentration were significantly lower than those in healthy children. •At 4 weeks post vaccination GMC of the NS group was lower for all strains; post vaccination combined GM was noted to be 67.4 and 43.1 for healthy children and children with NS, respectively. •Comparable percentage of children who attained a 2 fold or greater increase in the value of combined antibody concentration among the healthy and NS group at 66.6% and 68.75, respectively. •Type of NS (Steroid responsive or resistant) and the stage (remission or relapse) had no impact on the GMC.	(37)
Children with SSNS; 36 months.	2004	To investigate the degree of persistence of IgG antibody concentration at intervals for 3 years post vaccination.	Children with SSNS, *n* = 9	All participants, 3.51–9.02 years (mean: 6.25 years)	PPV23 given during remission period of at least 2.5 months while off corticosteroids	NIL	•IgG antibody titer increased by at least 2-fold from baseline antibody levels (between 4 and 86 mg/l) to reach a mean arithmetic value of 165.4 mg/l, 4 weeks after the administration of vaccine. •At the end of 36 months, 4/9 patients experienced the fall of antibody titer to below baseline or post vaccination values.	(38)
Children with INS, 18 months	2008	To show that there is a serological response to vaccine in nephrotic children at disease onset on high-dose prednisolone	Group 1 (vaccination during active disease, on high dose oral prednisolone therapy), *n* = 30; Group 2 (vaccination during remission, on low dose oral prednisolone therapy), *n* = 13; Group 3 (historical cohort with no vaccination but underwent same treatment protocol), *n* = 25	**Age of Disease onset;** Vaccine at Onset group: 2–16 years,Vaccine in Remission group: 2–9 years.**Age of vaccination**; Group vaccinated at Onset: 2–16 years,Group Vaccinated in Remission: 5.1–17 years	PPV23	High dose oral prednisolone: 60 mg/m^2^ to a maximum of 60 mg per dayLow dose oral prednisolone: ≤ 15 mg/m^2^	•Nephrotic children on high-dose glucocorticoid therapy respond to a 23-valent PPV Group 1 achieved a 10 fold increase in antibody concentration on day 30, from a mean of 1.3–11.3 μg/ml. Both groups 1 and 2 showed similar antibody response throughout the study duration of 18 months, particularly in the first month. •No difference between the course of disease of group 1 and 3 was identified.	(39)
Children with INS, 36 months	2010	Follow up from the previous study (39) to investigate if the antibody levels remains stable after 36 months post vaccination	Group 1 (vaccination during remission, on low dose oral prednisolone therapy), *n* = 8; Group 2 (vaccination during active disease, on high dose oral prednisolone therapy), *n* = 13	As for Ulinski (39) above	PPV23	As for Ulinski (39) above	•Antibody levels remained elevated in both groups 1 and 2 even after 36 months and no cases of invasive pneumococcal infection among the participants.	(40)
Children with INS, 6 months	2012	To investigate the effect of vitamin D on vaccine response in nephrotic patients.	Children with INS, *n* = 25	All participants, 5.3 +/– 2.39 years	PPV23	NIL	•No correlation was found between Vitamin D plasma levels and anti-pneumococcal antibody levels at baseline, 1, 3, and 6 months post-vaccination	(41)

*n = number of participants in the group that have completed the study*.

**Table 3:** Summary of trials of PCV efficacy in pediatric Nephrotic Syndrome population.

**Study group, follow up duration per patient**	**Study year**	**Primary aim**	**Number of subjects (Intervention group and Control/comparison group)**	**Age group (mean/ median)**	**Type of vaccine used**	**Steroids/ medications intake, dosage, duration (if applicable)**	**Results**	**Reference**
Children with INS, 12–14 months	2011	To assess the safety, immunogenicity, and kinetics of the immune response of 7 PCV in INS patients and healthy subjects.	33 patients with NS: Group A (on treatment of low dose oral prednisolone or no therapy), *n* = 15; Group B (on additional treatment of Mycophenolate mofetil or cyclosporine A for a minimum of 6 months prior to enrolment), *n* = 18; Control group consisting of patients' healthy siblings, *n* = 16	Group A, 8.8 +/- 1.8 years, Group B, 12.4+/- 3.7 years	PCV7 given during remission period and booster dose administered 12 to 14 months after priming	Group A: steroid therapy, <1 mg/kg, every other day or no therapy, Group B: Additional therapy with Mycophenolate mofetil and/or Cyclosporine A for 6 months.	•PCV 7 is safe and immunogenic in pediatric patients with INS in remission; No association of PCV7 with INS recurrence noted •At 1 month, a significant portion of group A, B and control (100%, 94% and 100% respectively) had antibody levels above the protective threshold of 0.35 μg/ml for ≥5 pneumococcal serotype (PS). GMC of antibodies for PS 4, 9 and 18C were significantly lower in Group B compared to controls. Group A results were similar to controls. •Most subjects retained protective antibody levels for ≥ 5 PS: 86%, 94% and 100% in group A, B and controls at 12- 14 months. When higher cut-off concentration of 1.00 μg/ml was used to interpret the data, controls demonstrated the greatest retention of protective antibody levels against ≥5 PS. •GMCs of all groups were above baseline levels. Group A showed significant reduction in antibodies for PS 4, 9v and 18c while group B demonstrated a similar reduction in antibody levels against PS 6B and 18 C.	(42)
Children with INS; 6 months	2014	Continuation of the previous study (42) to evaluate the safety profile and immunogenicity of the serological response of booster dose of PCV7.	Group A (on treatment if low dose oral prednisolone or no therapy), *n* = 14; Group B (on additional treatment of Mycophenolate mofetil or cyclosporine A for a minimum of 6 months prior to enrolment), *n* = 15	As above	A booster dose of PCV7 given after an initial dose 12 months ago	As above	•Additional PCV7 doses can be safely given to children with INS to increase circulation antibodies above the protective threshold. •Booster vaccination was not related to increased risk of INS relapse as compared with the 6 months before the administration of vaccine, *p* = 0.8. Post booster GMC s were comparable to the post primary vaccination responses. •GMC of antibodies increased remarkably (*p* < 0.01) following administration of booster vaccine compared to levels prior to administration of booster. •The highest GMCs were achieved for PS 14 and 23F, whereas PS 4, 9V, and 18C were the least immunogenic in both groups. When compared with Group B, Group A achieved higher GMCs for all PS, with PS 4, 9v and 18c in particular achieving significantly higher fold rises.	(43)
Children with NS (at onset or relapse or regular follow up); 1 year	2016	To assess the baseline seroprotection of NS patients against Strep. pneumonia as well as the safety, immunogenecity and how treatment affects the vaccine response.	Treatment free group, *n* = 20; Prednisolone only group, *n* = 7; Alternative immunosuppressive medication (AIM) only group, *n* = 7; Both prednisolone and AIM group, *n* = 8	All participants, 1–18 years(mean 7.7 years)	PCV13	Prednisolone, dosing, and duration not given, AIM, dosing and duration not given	•PCV13 immunization in NS children is highly immunogenic regardless of treatment and induces high serotype-specific IgG titers that are maintained in the high range 1 year after immunization •Post immunization, IgG titres rose significantly for all PS with an average of 2.8 out of 3 serotypes tested reaching high titres (>1 mg/L) at months 3 and 12 with persistently high titres at month 12. The vaccine response was noted to be the same for patients who were included and received PCV13 at disease onset as well as those who received PCV13 during follow up. •However, patients received prednisolone or AIM were noted to have lower serotype IgG titres for serotypes 14 and 23 F. This persisted for serotype 14 at 12 months follow up. 14% of patients immunized during follow up relapsed and all of this cohort had at least one relapse episode prior to vaccination.	(44)

*n, number of participants in the group that have completed the study*.

Overall, the data for PPV trials indicate that NS patients are able to generate antibody responses comparable to control groups, achieving protective levels of antibody with geometric mean titers of >200 ngN/mL, for all strains included except strain 19, especially 19F (6, 33–35). The lack of response to strain 19F may be an area worth researching further as it may offer insights into ways to enhance immunogenicity in the immunocompromised cohort.

Lee et al. (37) demonstrated that NS children were able to increase their antibody levels to 2x baseline, however compared to controls, their baseline antibody levels were lower hence final concentration of antibody was lower relative to healthy controls.

In studies which included a proportion of the cohort on steroids or immunosuppressives, the patients on these medications were able to achieve protective levels of antibodies following immunization with PPV (6, 34, 35) and PCV (42, 44).

Ulinski et al. (39) demonstrated that PPV23 given during active disease to patients on high dose prednisolone were able to mount an antibody response of a 10-fold increase on day 30 post vaccination, similar to that of NS patients given vaccine in remission while on low dose prednisolone. In a follow up to this study at 36 months post vaccination, antibody levels remained high in both groups and there were no reports of invasive pneumococcal disease in any of the participants (40). Results from Guven et al. (38) however, showed that 4 out of 9 patients with SSNS had a drop in antibody titers to below baseline by 36 months post PPV23 vaccination.

Persistence of antibody response after PPV 14 was maintained above protective levels 5 years after initial vaccination in NS patients with MCNS but not those with non-MCNS variants (35). Spika et al. (36) demonstrated persistence of antibodies above protective levels 1 year after initial vaccination in 50% of patients against 6 strains, however they found that antibody levels dropped faster in relapsers compared to non-relapsers.

Garin and Barrett (7) demonstrated that even NS patients in active disease are able to mount IgM response against serotypes 3 and 19 post PPV 14 or PPV23 at levels comparable to controls but had significantly lower IgG levels which may be attributed to urinary losses of IgG in active disease as IgG was found in the urine of NS patients and there was a significant correlation between albumin levels and antibody titer.

There is relatively less literature available for PCV as compared to PPV, but the data suggests that in NS patients, PCV7 is able to provoke a rise in baseline antibody levels to above the protective threshold, though the levels vary for different serotypes (42). At 12–14 months post vaccination, the protective levels of antibody were maintained above baseline. This cohort were followed up and it was demonstrated that booster PCV7 dose 12 months after initial dose in NS patients is safe and was able to provoke a significant rise in antibody levels (43).

PCV 13 has also been shown to be highly immunogenic in NS patients with the ability to induce high serotype-specific IgG titers that are maintained in the high range 1 year after immunization. This patient cohort included those on steroids and immunosuppressive and all groups had similar vaccine responses; however, those on prednisolone or immunosuppressives had lower IgG titers for serotypes 14 and 23F.

Overall the data seem to suggest that patients with nephrotic syndrome are able to mount an adequate immune response both to PPV as well as PCV even when the vaccine is given during active disease and while on steroids or immunosuppressives. However, in those with greater degrees of urinary losses, there appears to also be urinary losses of IgG resulting in reduced antibody titers. However, there is limited data on whether or not antibody titers can accurately be correlated to actual outcomes in terms of actual reduction of pneumococcal infections clinically.

## Safety of Pneumococcal Vaccination in Children With Nephrotic Syndrome

Pneumococcal vaccination has generally been shown to be safe by numerous studies. Local reactions appear to be one of the few common problems; these are a relatively minor complication which are not of great clinical significance. In 1978, Fikrig et al. had established that vaccination of NS patients and healthy controls with PPV13 did not lead to any significant adverse effects, with the only side effects noted being local soreness (33). Lee et al. (37) had demonstrated similar findings with PPV23, with 38% of children with NS and 50% of subjects in the healthy group developing local reactions to it; in this study, the probability of patients with NS developing local reactions appears to be lower than healthy subjects (37). Pittet et al. however, found that local and systemic reactions to PCV13 among children with NS were found to be comparable to healthy children. More importantly, no serious adverse events were recorded in this study; and no association was found between vaccination with PCV13 and relapse of NS. Among 8 patients who experienced at least one relapse during 1 year of follow up, 3 were vaccinated at disease onset while 5 received PCV13 during follow up. The relapse risk attributed to NS itself for this group of patients was 1 relapse per year, whereas the corresponding value for the patients vaccinated at disease onset was not known. The occurrence of relapse appears to have remained unchanged post-vaccination for patients vaccinated during follow up, indicating that PCV13 on its own does not increase relapse risk (44). Liakou et al. too reported that local and systemic reactions recorded within 7 days to PCV7 were similar in patients and controls. More specifically, reactions involving only the injection site seemed to be commonest with 30.8% of all vaccinated individuals experiencing them. In contrast, only 2% of all vaccinated individual developed fever and hence, appears to occur less frequently than injection site reactions. Administration of a booster of PCV7 was also noted to cause similar rates of systemic reactions, injection-site adverse reactions along with fever (43). In addition, this study also provided evidence that vaccination with PCV7 does not contribute to an increased risk of relapse, with a total of 20 episodes of relapse occurring a year prior to vaccination while only 10 such episodes occurred post-vaccination (42). The administration of booster vaccination was not associated with an increased risk of relapse (43).

A case report describing the emergence of a *Pityriasis rosea* like eruption, in a child with NS who had received a synthetic Hepatitis B and a Polyvalent conjugate vaccine (PCV) was noted. This eruption was thought to be caused by PCV due to the strong correlation between the time of PCV administration and eruption, and the absence of recurrence of eruptions when the patient received further doses of Hepatitis B vaccine (66). Although this might be an isolated case, it does demonstrate to the possibility of the occurrence of *Pityriasis rosea* like eruption with PCV administration.

Overall, there is a reassuring lack of serious adverse effects reported to the pneumococcal vaccination; and there appears to be a similar side effect profile in children with NS and healthy controls. Therefore, the considerable benefits described above, on a background of *S. pneumoniae* being the commonest cause of infection in children with INS (29), would significantly outweigh the relatively mild side effects of PCV and PPV23. Consequently, pneumococcal vaccination in children with INS is to be encouraged.

Among the studies that were reviewed, numerous studies were used enzyme linked immunosorbent assays (ELISA) to measure serotype-specific antibodies (39, 40, 42–44), whereas older studies had measured total anti-pneumococcal antibody titres. A potential drawback of this method would be the detection of serotype specific non-opsonic antibodies, which do not confer any form of protection. Hence, it is not as effective as opsonophagocytosis assay (OPA) in predicting the true level of protection provided by the antibodies. However, it is important to take note that ELISA has received extensive validation due to the emergence and establishment of antibody thresholds that provide adequate protection against Invasive pneumococcal disease among children (67). Furthermore, the strong correlation between OPA titres and ELISA IgG levels in children provides further strengthens the validity of ELISA. However, the concordance between OPA titres and ELISA IgG levels is less prominent among adults (68).

## Barriers to Vaccination of Nephrotic Patients

Key barriers to pediatric NS patients receiving the pneumococcal vaccine would be the prohibitively high cost and the fact that a great deal of confusion surrounds the optimal vaccination protocol in NS patients.

### Cost as a Barrier to Vaccination

Not all countries have introduced pneumococcal vaccination as part of their national immunization program. Even in countries where it is recommended, it is not always subsided or funded by the government, making it prohibitive for the average citizen to have their children vaccinated. A total of 142 countries have introduced PCV from 2000 through 2018. Of the 73 Gavi-eligible countries, 59 (81%) introduced PCV. Among non-Gavi eligible countries, PCV has been introduced in 6 (50%) of 12 lower middle-income countries, 26 (51%) of 51 upper middle-income countries, and 51 (88%) of 58 high-income countries (57). Given the prohibitively high cost of the vaccine in the private sector, government support would be including pneumococcal vaccination in the NIP would greatly boost uptake.

There was no specific data available for cost effectiveness of pneumococcal vaccinations of PCV vs. PPV in the pediatric nephrotic population, however a report by ACIP (2012) looking at cost-effectiveness of immunizing immunocompromised adults against pneumococcal disease with regimen of PCV13 at time of diagnosis followed by current PPV 23 vaccination guidelines starting 1 year later resulted in a cost saving of $7,600,000 and added 1360 quality adjusted life years and averted 57 cases of invasive pneumococcal disease.

Cho et al. (69) reported that adding a single dose of PCV 13 as an additional dose to immunocompromised adults instead of only the recommended PPV 23 potentially reduces both disease and costs. The additional cost of giving the additional dose of PCV 13 would cost $16 million (in 2009$) but provide off-setting savings of $21 million per cohort from the societal perspective. This dose of PCV13 would prevent 57 cases of invasive pneumococcal disease, 619 cases of hospitalized all-cause pneumonia, avert 93 deaths, and save 1360 quality adjusted life years per cohort. While this is for an adult population and covered 4 immunocompromising conditions, it seems logical that to extrapolate that this should be applicable to the pediatric nephrotic population as well (particularly those >2 years old who may only be recommended to receive PPV).

Based on the data currently available, it seems to suggest that vaccination against pneumococcal disease in NS patients is likely to be cost-effective in the long run.

### Lack of Clarity Regarding Protocols for Pneumococcal Vaccination

Among nephrotic patients specifically there is a great deal of uncertainty among medical practitioners regarding appropriate vaccination practices—whether or not is appropriate and effective, and even if they feel it is appropriate, there is uncertainty regarding dose and timing. Clear guidelines should be drawn up at national or institutional level and then implemented. Given that the majority of pediatric patients with NS will end up admitted to a tertiary level pediatric facility, particularly at first diagnosis, this represents an opportunity to screen vaccination status and administer appropriate vaccines.

Smith and Metzger (70) reported the success of a multifaceted intervention on pneumococcal vaccine in screening and administration rates in eligible patients who were admitted to an internal medicine unit of a tertiary care teaching hospital—their interventions included a revised nurse screening tool, rescheduling of the vaccine order, storage of the vaccine in automated dispensing cabinets on the nursing unit and creation of a vaccine tracking system. The rate of pneumococcal vaccine administration in eligible patients significantly improved post-implementation compared with pre-implementation (74.2 vs. 19.1%, respectively, *P* < 0.001). Harris et al. (71) also reported a similar improvement when a multifaceted protocol including clear guidelines and procedure for screening and flagging patients for whom pneumococcal vaccine was indicated was introduced in a pediatric rheumatology unit. The authors note it was relatively simple to institute and maintain this system in a well run hospital unit, making it a model that could be used in pediatric units managing NS patients to help optimize pneumococcal vaccination practices. Clear guidelines need to be drawn up regarding the pneumococcal vaccination policy in NS patients and they could then be implemented through a similar model.

## Limitations and Future Directions

Since their introduction in 1977, pneumococcal vaccines have been used for the prevention of invasive pneumococcal diseases, which have high prevalence and mortality rate among nephrotic children. The data supporting the use of these vaccines and to guide the timing and dosage recommendations is however, limited.

Although several studies have been conducted they are limited by small sample size, ranging from 9 to 68 participants (38, 39). A larger number of participants is required to produce results with adequate statistical power, however, due to ethical considerations and complexity of trial design when involving high risk and immunocompromised patients, such trials are extremely difficult to be carried out. Nevertheless, more focus should be put on conducting international, multicentre randomized controlled trials which involve a large sample size in the future, with particular emphasis on establishing the efficacy of the pneumococcal conjugate vaccine for which the evidence up to today, is very much lacking.

In addition, controversial results have been reported on whether or not antibody titer correlates with clinical efficacy of the vaccine in nephrotic patients (35, 72). We feel that this is an important area for future work as all studies to date measure antibody concentration rather than the clinical efficacy of the vaccine directly, i.e., risk of pneumococcal peritonitis. The vaccine will not serve its intended purpose if it induces an increase in antibody titers but does not provide adequate anti-pneumococcal protection.

Compared to PPV, PCV is a much more recent invention and only a handful of trials have been conducted on it. Nevertheless, guidelines recommend the use of PCV in nephrotic children as PPV in some studies shows inconsistency in its immune activity in children below 2 years old as described previously. Thus, we suggest future work to focus on the study of PCVs compared to PPV; there is still much yet to be explored for PCV, for example the persistence of antibody concentrations after a period of time, its effectiveness in different types of NS and factors that may affect its activity such as high dose steroid treatment, the use of alkylating agents, plasma albumin level and stage of disease where vaccine is administered. Endeavors can also be made to increase the cost effectiveness of the production of PCV as the costly vaccine up until now, is unaffordable to many.

Although perhaps less in vogue now, research on PPV is still clinically relevant as it is still frequently used, especially in patients above 2 years old. The explanation for poorer antibody response toward PS19 is inadequate and it is important to know the reason since PS19 is commonly involved in pneumococcal diseases in individuals in all age groups (7). Type specific analysis was suggested to study the fall in antibody titres by Spika et al. as it was found to differ by capsular type (36). Participants in the study by Ulinski et al. demonstrated different increase of post-vaccination antibody titer from a range of 2–50 folds, and after exploring various possible factors such as age, plasma albumin level, steroid treatment and even vitamin D level, the cause behind was still not known. This unanswered research question provides an opportunity for future studies to be carried out and the information yielded from these studies could prove to be decisive in the development of a new and improved version of the vaccine (39).

## Conclusions

After review of the literature, we would recommend that all children be immunized against *S. pneumoniae* and agree strongly with the WHO recommendation that pneumococcal vaccination be made part of all national immunization programs. This would then confer protective immunity against *S. pneumoniae* in the vast majority of children who develop NS as this usually only first presents between the ages of 2 and 6. Even in children who have completed their primary series, given that NS is an immunocompromising and therefore high risk condition, we would suggest an additional dose of PPV23 at point of diagnosis even though the patient is commencing steroid therapy as there still appears to be an adequate response in active disease and on steroids. For NS patients aged > 2 years old who have never been immunized against pneumococcus, we would recommend a primary dose of PCV 13 followed by PPV23; again we would suggest that the vaccination be given early in the disease process as the studies seem to indicate an adequate antibody response, though need for subsequent boosters may need to be guided by the severity of protein loss as well as other clinical factors.

## Author Contributions

The writing was performed by SG, CT, LT-HT, PL, PP, L-HL, and B-HG. While, PP, PL, L-HL, K-GC, and B-HG provided vital guidance and insight to the work. The project was conceptualized by B-HG and PP.

### Conflict of Interest Statement

The authors declare that the research was conducted in the absence of any commercial or financial relationships that could be construed as a potential conflict of interest.
